# Self-harm and Violence Presenting to Emergency Care Registry (SAVER) project: protocol for a mixed-methods study

**DOI:** 10.1136/bmjoq-2025-003463

**Published:** 2025-09-14

**Authors:** Emily Bebbington, Anne Krayer, Abigail Lea, Alberto Salmoiraghi, Claire Cotter, Deborah Job, Gemma Hobson, Geraint Farr, Gwennan Charlton, Jane Moore, Limssy Varghese, Meinir Evans, Ned Hartfiel, Non Evans, Rebecca Masters, Rhiannon Tudor Edwards, Robert Atenstaedt, Rob Poole, Rosalind Reilly, Catherine Robinson

**Affiliations:** 1 Centre for Mental Health and Society, Bangor University, Wrexham, UK; 2 Public Health Wales NHS Trust, Cardiff, UK; 3 Data Intelligence and Insight Department, Betsi Cadwaladr University Health Board, Bangor, UK; 4 Mental Health and Learning Disabilities, Betsi Cadwaladr University Health Board, Wrexham, UK; 5 Suicide and Self-harm Prevention, NHS Wales Executive, Cardiff, UK; 6 Public Health, Aneurin Bevan University Health Board, Newport, UK; 7 Unscheduled and Emergency Care, Betsi Cadwaladr University Health Board, Bangor, UK; 8 Public Health, Betsi Cadwaladr University Health Board, Bangor, UK; 9 Centre for Health Economics & Medicines Evaluation, Bangor University, Bangor, UK; 10 Canolfan Felin Fach, Pwllheli, UK; 11 Emergency Department, Ysbyty Gwynedd, Bangor, UK; 12 WHO Collaborating Centre on Investment for Health and Health Well-being, Public Health Wales NHS Trust, Cardiff, UK; 13 Data Knowledge and Research Directorate, Public Health Wales NHS Trust, Cardiff, UK; 14 Social Care and Society, The University of Manchester, Manchester, UK

**Keywords:** Electronic Health Records, Emergency department, Process mapping, Qualitative research, Registries

## Abstract

**Introduction:**

Intentional injuries can be broadly classified into those that are self-inflicted (eg, suicide, self-harm), and those that are inflicted by others (eg, homicide, assault). Many risk factors are the same for all intentional injuries. It is widely accepted that there needs to be a public health approach to self-harm and interpersonal violence prevention, including surveillance of presentations to emergency departments. Self-harm and interpersonal violence are important causes of morbidity and mortality in Wales. Interpersonal violence surveillance is already operationalised in Wales, but variables are limited and case ascertainment may not be complete. There is no self-harm register. The aim of this study is to understand the utility of existing systems in North Wales that collect data about self-harm and interpersonal violence, and how a registry could be implemented to address any unmet needs.

**Methods and analysis:**

The project consists of five work packages. First, process mapping will be used to understand the pathways by which patients access emergency care, and how data are collected about patients. Second, routinely collected data will be explored to understand the burden of disease, and the strengths and limitations of existing data collection systems. Third, semi-structured interviews will be completed with stakeholders to understand their needs and experiences. Fourth, semi-structured interviews with third sector organisations which work with people with lived experience of self-harm or interpersonal violence will explore the acceptability of data collection. Fifth, a method will be developed that would enable economic evaluation of a self-harm and interpersonal violence register.

**Ethics and dissemination:**

Results will be used to understand whether a self-harm and interpersonal violence registry is required in Wales. The results have the potential to influence local and national strategy on intentional injury prevention. Results will be disseminated to local services, regional and national programme teams, and published as a peer-reviewed journal article.

**WHAT IS ALREADY KNOWN ON THIS TOPIC**

- This study will directly examine the interrelationship of self-harm and interpersonal violence—issues often treated in isolation—providing a foundation for a unified public health approach to prevention.

**WHAT THIS STUDY ADDS**

- A broad range of quantitative and qualitative data will be synthesised to understand the utility of existing systems that collect data about self-harm and interpersonal violence, and how a registry could be implemented to address any unmet needs.

**HOW THIS STUDY MIGHT AFFECT RESEARCH, PRACTICE OR POLICY**

- Development of a self-harm registry in Wales has been recommended for over 15 years in the national suicide and self-harm prevention strategy. This study will directly address that unmet policy need.
- The methods are likely to be of wider interest to stakeholders seeking to evaluate and enhance data systems for the prevention of self-harm and interpersonal violence.

## Introduction

### Background

 Intentional injuries can be broadly classified into those that are self-inflicted (eg, suicide, self-harm) and those that are inflicted by another individual or small group of individuals (eg, homicide, assault).^1^ The WHO definition of violence highlights that physical force against oneself or others can result in physical injury, psychological harm and deprivation.^2^ This may be acute, such as requiring emergency care at a hospital for a physical injury; or latent, such as an increased risk of future self-harm or involvement in a violent crime for those who have faced multiple adverse childhood experiences.^3^ The HM Government Serious Violence Strategy and national suicide prevention strategies in the UK recognise that interpersonal violence increases the risk of suicide and self-harm, and that there are common risk factors including poverty, substance misuse and social isolation.^4^,^6^ There is an increasing recognition of the need for a public health approach to self-inflicted and interpersonal violence prevention.^7^,^9^ Key to this is the availability of timely and reliable data to inform service developments and upstream preventative interventions (table 1).

**Table 1:** Examples of how timely self-harm and interpersonal violence data from emergency department presentations could be used to inform upstream preventative interventions

Output	Hypothetical finding	Example upstream intervention
Identification of high-risk groups	Higher rates of self-harm among females aged 15–24	School-based mental health programmes
Peak presentations for injury types	Increased alcohol-related violent injuries 22:00–03:00 at weekends	Informing alcohol licensing arrangements
Geographic hotspots	Increased violent injuries at certain locations	Safer environmental design such as improved street lighting
Escalating risk	Individuals with increasing number of presentations and injury severity	Multi-agency safeguarding review and appropriate community support
Emerging methods	Spike in use of self-harm packs bought from the internet	Policing restrictions on culprit websites
Social determinants	Higher rates of violence-related injuries among homeless individuals	ED referral pathways for housing and employment support

ED, emergency department.

### Need for clinical registries collecting data on self-harm and interpersonal violence

#### Self-harm

Self-harm is a broad term that captures all non-fatal self-inflicted poisonings and injuries, irrespective of the intention to die.^10^ Self-harm is the strongest predictor of future suicide.^11^,^14^ Target 3.4 of the United Nations Sustainable Development Goals is to reduce global suicides by one-third by 2030.^15^ The WHO recommends the systematic collection of data on self-harm as a key governmental action for suicide prevention.^11 16^ These data should include all cases of self-harm that present for emergency care to hospital, meaning data should typically be collected from emergency departments (EDs).^16^ Data may be collected using a dedicated register, national routine databases, or in individual studies.^16^ Dedicated registers are likely to provide the most tailored and long-term data that can be used to inform self-harm and suicide prevention strategies, track emerging trends, monitor the effectiveness of interventions and provide information that can form the basis for service changes.^16^

In the UK, there are dedicated ED-based self-harm registers in England and Northern Ireland.^17 18^ Data from these registers have been invaluable for informing policy, such as restriction of paracetamol and salicylate sales.^19^ There is currently no dedicated self-harm register in Wales or Scotland, though as part of Scotland’s new Self-Harm Strategy and Action Plan, data about self-harm presenting to secondary care will be reviewed and improved.^20^ The ‘Talk to Me 2’ cross-governmental suicide and self-harm prevention strategy for Wales 2015–2022, and the preceding plan for 2009–2014, recommended that there should be a self-harm register in Wales.^21 22^ The 2023 ‘Talk to Me 2’ end-point review showed that stakeholders believe that there is a data gap for self-harm that needs to be addressed.^23^

#### Interpersonal violence

The WHO subdivides interpersonal violence into two main categories: family and intimate partner violence (eg, child maltreatment, intimate partner violence, elder abuse); and community violence (eg, youth violence, assault by strangers, violence in workplaces).^24^ Four of the Sustainable Development Goal targets (SDG 5.2, 5.3, 16.1 and 16.2) relate to the reduction of interpersonal violence, highlighting the importance of violence prevention in order to ensure health and justice for all.^25^ The WHO Violence Prevention Unit recommends surveillance as a key step in a public health approach to violence prevention by enabling the definition of the problem through systematic data collection.^26^ This should include fatal (ie, homicide) and non-fatal (ie, assault) data.^26^

EDs are a key data collection point for non-fatal data, as it is well established that much interpersonal violence is not reported to the police, making EDs an important data source and potential intervention point.^27^ The 2022 Serious Violence Duty in England and Wales requires health services to share data on violent injuries treated in National Health Service (NHS) urgent care settings to support needs assessments and preventative interventions.^8^ The recommended anonymised data set follows The Cardiff Model’s Information Sharing to Tackle Violence (ISTV) minimum data set, which is now collected across all EDs in England and Wales and shared with Community Safe Partnerships (eg, police, fire and rescue) to help reduce serious violence.^8 28 29^

Routinely collected data on presentations of interpersonal violence to EDs in England and Wales is now regularly reviewed—an important advancement in surveillance that currently surpasses efforts for monitoring self-harm presentations in Wales. The Wales Violence Prevention Unit data set for EDs, based on ISTV, is limited to 12 surveillance-focussed variables, and not all forms of interpersonal violence (eg, domestic violence) are comprehensively captured.^30^ Additionally, differentiating injury intent is complex and prone to misclassification bias, which can affect the reliability of the data.^31^ Given these limitations, it would be beneficial to explore existing data sources for both self-inflicted and interpersonal violence to assess opportunities for improved case ascertainment and whether more detailed variables could be collected to inform local services and strengthen violence prevention efforts. This may lead to improved data capture in EDs that could be used locally as well as feeding into wider surveillance by the Wales Violence Prevention Unit.

### Existing data sources in Wales

Self-inflicted and interpersonal violence are major causes of morbidity and mortality in Wales. In 2023, there were 386 registered suicides in Wales (14.0 deaths per 100 000 people), and in the year ending March 2023, there were 23 homicides (3-year rate of 0.79 per 100 000 people).^32 33^ There were over 5000 admissions for self-harm in Wales in 2022/2023, of which approximately a third were in North Wales (table 2).^34 35^ Admissions for violence are lower, with approximately 700 in Wales in 2022/2023.^34 36^

**Table 2:** Admissions to Welsh hospitals by Welsh residents and non-Welsh residents for self-harm, assault, and undetermined intent in 2023/2024^36 51^

Health board	Self-harm	Assault	Undetermined intent
Aneurin Bevan University Health Board	679	125	–
Betsi Cadwaladr University Health Board	1775	130	6
Cardiff and Vale University Health Board	359	61	–
Cwm Taf Morgannwg University Health Board	712	115	–
Hywel Dda University Health Board	778	22	–
Powys Teaching Health Board	12	–	12
Swansea Bay University Health Board	773	219	3
All Wales	5100	686	54

Data produced by Digital Health and Care Wales using Admitted Patient Care (APC) data. Data collated for ICD-10 external cause codes for self-harm (X60–X84), assault (X85–Y09), and undetermined intent (Y10–Y34).^52^ Please note that the number of admissions is underestimated in each cell of the table because ICD-10 codes with fewer than three patients have suppressed values.

Quoted data for hospital presenting cases of self-harm or other forms of violence in Wales typically relate to hospital admissions (captured in the Admitted Patient Care dataset, APC), rather than ED attendances (captured in the Emergency Department Data Set, EDDS).^6 37 38^ Hospital admission data from APC are likely to underestimate the true burden of disease because not all patients require admission that present with self-harm or interpersonal violence. This was shown in a study of 10–24 year olds in Wales using EDDS and APC data from 2003 to 2015, in which less than half of ED attendances for self-harm resulted in a hospital admission.^39^ A similar study of adults has not been conducted in Wales. In England, routinely collected hospital admissions data have been shown to underestimate admissions for self-harm by approximately 40% compared with dedicated self-harm registers.^40^ Admissions data relies on timely ICD-10 coding, but recent staffing and resource constraints in NHS Wales Clinical Coding have led to reduced coding completeness (ie, increases in missing data), creating the appearance of declining cases when this is actually an artefact.^41^

Missing data are a concern with ED data and have the potential to give misleading results that may affect service changes.^42^ To improve the quality and consistency of information collected across urgent and emergency care pathways in Wales, EDDS will soon be replaced by the Welsh Emergency Care Data Set (WECDS).^43^ WECDS has been adapted from the Emergency Care Data Set that has been in use in NHS England since 2023.^43^ EDDS is a basic data set completed for every presentation to an ED in Wales. However, the data are not routinely published.^38^ Routinely collected ED data may underestimate the true number of presentations due to inaccurate coding. In England, routinely coded records of presentations of self-harm underestimated the number of cases by approximately 60% compared to a dedicated self-harm register with a properly designed case ascertainment strategy.^40^ EDDS is used as the basis for surveillance of interpersonal violence by the Wales Violence Prevention Unit and it is recognised that there is likely to be under-ascertainment.^30^ It is not currently understood whether these data could be used, or augmented, for routine surveillance of self-harm and improved surveillance of interpersonal violence. The utility of existing data sources, including EDDS (and WECDS in the future) and APC for a self-harm and violence register in Wales needs to be explored further.

### Unified approach

There is a need for the collection of high-quality data on presentations for self-harm or interpersonal violence. Since self-harm and other forms of violence share many antecedents, a unified registry could efficiently collect and monitor data to inform services, helping reduce the siloing of public health approaches to intentional injury prevention. A register can use existing routinely collected data, or a bespoke parallel data collection process. In order to determine the most appropriate solution, it is essential to understand what routinely collected data is available, how these data are collected, and the needs of stakeholders. There are multiple means by which patients presenting with self-harm and interpersonal violence can gain emergency care, including via ambulance, minor injury units (MIUs), EDs, and direct interaction with specialty services (eg, psychiatry). We do not currently understand the different patient pathways, number of presentations at each point, strengths and limitations of existing routinely collected data, needs of service users, or stakeholder needs. These data are essential to determine how a self-harm and violence register might be implemented, and how the data might be best used by stakeholders.

### Aim and objectives

The aim of this project is to understand the utility of existing systems in North Wales that collect data about self-harm and interpersonal violence, and how a registry could be implemented to address any unmet needs.

The objectives of the project are to:

1. Understand the pathways by which patients gain emergency care in North Wales when presenting with self-harm or interpersonal violence, and how data are collected about patients in each pathway.
2. Explore existing data to understand the burden of disease, and strengths and limitations of existing data collection systems.
3. Understand stakeholder needs and experiences of use of self-harm and interpersonal violence data.
4. Work with third sector organisations who work with people with lived experience of self-harm or interpersonal violence to understand experiences of data collection, acceptability of data collection methods, and utility of the data.
5. Develop a method that would enable economic evaluation of a self-harm and interpersonal violence register.

## Methods and analysis

### Setting

Healthcare and public health services in Wales are delivered by seven NHS health boards.^44^ The largest health board in Wales is Betsi Cadwaladr University Health Board (BCUHB), which serves the North Wales region. North Wales has a population of approximately 700 000 people—just over a fifth of the total population of Wales.^45^ There are seven MIUs and three EDs in North Wales: Ysbyty Gwynedd, Bangor; Ysbyty Glan Clwyd, Rhyl; and Wrexham Maelor, Wrexham.^46^ The setting was chosen due to the existing networks of the authors, varied urgent and emergency care facilities that are representative of the variety of options available to the population of Wales, diverse geography, and mix of rural and urban centres. This protocol has been pre-registered on the Open Science Framework (https://doi.org/10.17605/OSF.IO/9BRMJ).

### Objective 1—Understand the pathways by which patients gain emergency care when presenting with self-harm or interpersonal violence, and how data are collected about patients in each pathway

Process mapping will be used to understand the pathways by which patients gain emergency care following self-harm or interpersonal violence. Methods developed for the establishment of self-harm registries will be followed and adapted to also incorporate data collection for those presenting following interpersonal violence.^47^ Interviews with key staff members will be undertaken to understand the processes that patients follow to gain emergency care. Staff will be recruited to understand the pathways at all points from presentation to discharge (eg, receptionists, triage nurses, ED doctors, psychiatric liaison nurses). Interviews will gather information to understand the points at which patients can make choices, triage processes, administrative procedures (eg, registration), options for patient disposition, and documentation. Interviews will initially be completed at the three major EDs and extended to MIUs if resources permit. Staff will be invited to participate in an interview by a gatekeeper (ie, senior staff member collaborating in the project). A participant information sheet will be provided and written informed consent will be taken. All information will be available in English and Welsh. Participants will be free to withdraw at any point. Interviews will be conducted at a time and location convenient to the interviewee. Interviews are likely to occur in the ED, and thus recording is unlikely to be appropriate. Comprehensive field notes will be taken and then written up and anonymised to maintain confidentiality of the participant. Data will be analysed to understand the processes followed at each point in the patient journey. An iterative process will be followed to generate narrative and pictorial process maps.^47^ No patient data will be collected.

### Objective 2—Explore existing data to understand the burden of disease, and strengths and limitations of existing data collection systems

Anonymised routinely collected data will be examined on ED presentations, inpatient admissions, and ambulance conveyances for self-harm and other forms of violence. ED and MIU data for presentations of self-harm or interpersonal violence will be identified from across North Wales using EDDS codes (eg, Patient Group: Deliberate self-harm) and keyword lookups (eg, “overdose”) in narrative text sections. The relative utility of the EDDS codes and keyword lookups will be compared. Inpatients will be identified using ICD-10 external cause codes (eg, X60-X84: Intentional self-harm, X85-Y09: Assault). Reliability of the codes will be assessed by comparison of results to a broader set of inclusion criteria that includes all injuries, poisonings, and overdoses irrespective of classification of intent. Completeness of the fields will be assessed.

**Figure 1:**
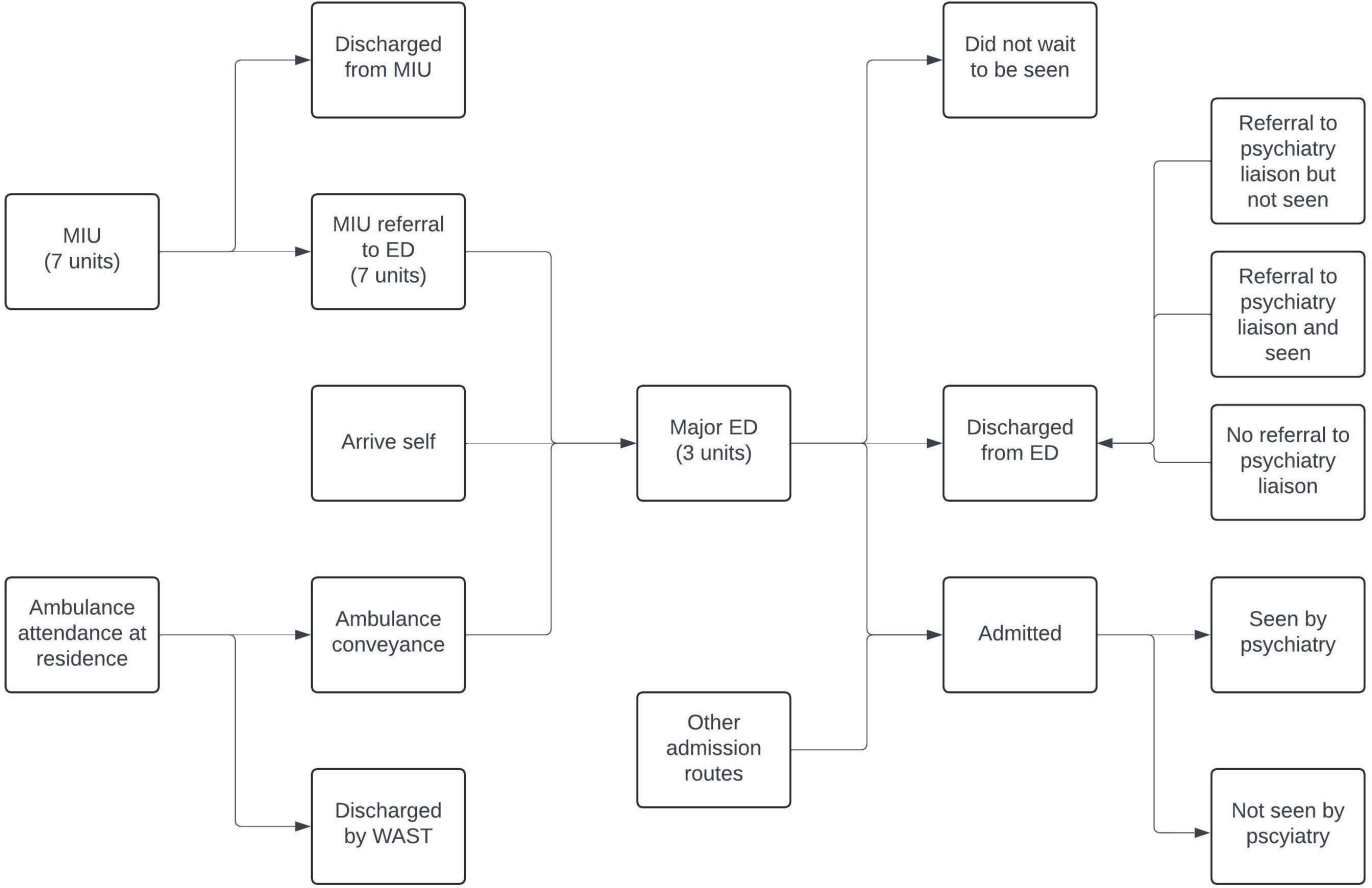
Schematic of the pathways to care for which routinely collected data will be analysed. ED, emergency department; MIU, minor injury unit; WAST, Welsh Ambulance Service.

Anonymised data will be sought by information analysts working in BCUHB. Descriptive data will be presented according to time (eg, time of presentation, day of week, month, year), person (eg, age, gender) and place (eg, emergency care site). This will be generated to understand the patient population at key points at which patients receive emergency care. These data will be combined with the process map from objective 1 to provide a fuller picture of the completeness of existing data sources and to understand the patient population at different points in the emergency care process (figure 1). It is likely that the quantitative data will include the number/proportion of patients:

- Conveyed by ambulance to MIU/ED.
- Presenting to MIU and that are subsequently seen in a major ED (Ysbyty Gwynedd, Ysbyty Glan Clwyd, Ysbyty Wrexham Maelor).
- Presenting to MIU and are not seen in a major ED.
- Do not wait to be seen by a doctor in MIU/ED.
- Referred to psychiatric liaison services.
- Admitted as an inpatient from MIU/ED.
- Admitted as an inpatient by other routes than MIU/ED.

### Objective 3—Understand stakeholder needs and experiences of use of self-harm and interpersonal violence data

Semi-structured interviews will be conducted with key stakeholders and knowledge users who could influence, be affected by, or benefit from the project.^48^ Interviews will focus on understanding their experiences of accessing and using data. Their data needs will also be explored to identify variables that would be useful to stakeholder groups in real time to improve services (eg, key performance indicators for EDs) and inform preventative interventions (eg, locations of concern, and targets for restriction of access to means). Different stakeholder groups will be recruited, including policy makers, service planners, health and care professionals, and those involved in multi-agency responses, to provide a broad overview of experiences and requirements (table 3). Further participants will be recruited through the existing network of stakeholders (ie, snowballing). A maximum variation sampling strategy will be employed to capture a broad range of perspectives, with an estimated 20 semi-structured interviews planned. All participants will be provided with information sheets prior to the interview and written informed consent will be taken. Participants will be free to withdraw at any point. Interviews will be conducted at a time and location convenient to the interviewee by a member of the research team with training in qualitative research methods. Notes will be taken and participants will be asked if they consent to the interview being recorded. Data will be transcribed and anonymised to maintain confidentiality of the participant. Data will be analysed thematically to understand the experiences and needs of different stakeholder groups.^49^

**Table 3:** Key stakeholder and knowledge user groups that will be approached to participate in a semi-structured interview

Group	Example organisation and target user groups
Policy makers	Welsh Government (eg, suicide prevention team)
Service planners	NHS Wales Executive (eg, suicide and self-harm prevention team)
Health and care professionals—national	Public Health Wales (eg, violence prevention unit team)
Health and care professionals—local	Betsi Cadwaladr University Health Board (eg, emergency medicine, psychiatry, public health services teams)
Other agencies	Police, local authority

NHS, National Health Service.

### Objective 4—Work with third sector organisations who work with people with lived experience of self-harm or interpersonal violence to understand experiences of data collection, acceptability of data collection methods, and utility of the data

Semi-structured interviews will be conducted with individuals working for third sector organisations that work with people with lived experience of self-harm or interpersonal violence in BCUHB. These interviews will focus on their views and professional experiences of local services (including secondary care) for self-harm, suicide, and interpersonal violence prevention; their perceptions of important data items; and how data could be used to enhance services. Given the sensitive nature of the topic, individuals will not be asked to share any personal experiences, but instead to use their professional experience to inform their recommendations. Local charitable organisations will be contacted to see if they wish to participate in the project. Methods of conduct and analysis of the interviews will be the same as those outlined in objective 3. We anticipate that approximately 10 interviews will be conducted.

### Objective 5—Develop a method that would enable economic evaluation of a self-harm and interpersonal violence register

A systematic scoping review will be conducted to identify current methods for the economic evaluation of clinical registries in secondary care (protocol published separately).^50^ Consultations with custodians of existing registries will be conducted to understand cost components and measurable outcomes. This review and consultation process will provide the basis for estimating direct and indirect costs associated with establishing and maintaining a self-harm and violence registry, accounting for factors such as staffing, training, and technological requirements.

To assess the potential benefits of the registry, we will adapt evaluation methods identified in the systematic scoping review and incorporate stakeholder recommendations on metrics critical for improving patient outcomes and prevention strategies (from interviews conducted for objectives 3 and 4). For example, anticipated benefits may include real-time feedback of data to services on intervention use, potentially reducing hospital length of stay and generating NHS cost savings. If the registry is implemented, these proposed methods could serve as an evaluation framework to assess economic viability and its justification as a public health investment.

### Patient and public involvement

A representative from a third sector organisation that supports individuals with lived experience of self-harm and interpersonal violence contributed to the design of this study (author: ME). Third sector organisations involved in objective 4 of the study will be invited to participate in a stakeholder workshop. This will focus on discussion of future research plans considering the study results. We aim to foster collaborations to co-design research for the next stages of this work.

## Ethics and dissemination

### Ethical considerations

This project is being conducted to understand current services in North Wales as supplied by Betsi Cadwaladr University Health Board (BCUHB) and how these could be improved in the future. The project is registered with BCUHB Clinical Effectiveness Team (Project Number: 1890), and as a service evaluation project with Bangor University Medical and Health Sciences Academic Ethics Committee. A data security and confidentiality agreement for BCUHB data has been completed by the project chief investigator. Retrieval of routinely collected data and recruitment to qualitative interviews is in progress. It is anticipated that data collection will be completed in May 2025.

### Limitations

There are some limitations to the proposed study. First, the work packages are restricted to a single region of Wales that has public health and healthcare provided by a single health board. The findings may not be generalisable to the whole of Wales. Second, it may not be possible to achieve data saturation in the qualitative interviews due to resource constraints such as availability of participants and short timescale of the project. Maximum variation sampling will be used to ensure that a wide range of views is captured. Third, we recognise that there are other categories of violence, including legal intervention, war, civil insurrection and disturbances.^1^ These are not a major cause of morbidity and mortality in Wales and thus are beyond the scope of this project. Fourth, the classification of injury intent is prone to misclassification bias, which is a potential limitation of any self-harm or interpersonal violence registry. Inclusion of both self-inflicted and violence-related injuries should enable exploration of this phenomenon. It will also be important to scrutinise injuries that might otherwise be coded as ‘unintentional’ to ensure accuracy and rigour of the eventual registry. Finally, we recognise that injuries due to self-harm or interpersonal violence may not always present to urgent and emergency care services (ie, ambulance and hospital). They may be cared for by other services in the community, including third sector, primary care and prison health services. Understanding the feasibility of establishing a surveillance system in the community is beyond the scope of this project. Any amendments to the study protocol will be reported in the final publication.

### Dissemination

The results from objectives 1–5 will be presented to project partners, stakeholders, and third sector organisations. Discussions will be held to understand whether existing systems to use data about self-harm and interpersonal violence meet stakeholder and service user needs. The results from the study will form the basis of future work to determine the most appropriate method to implement a self-harm and interpersonal violence registry in secondary care in Wales, and the organisations that could benefit from data sharing. Consideration will be given to financial implications, including the ability to calculate return on investment from a register itself. Conducting a regional pilot in North Wales may be the most viable approach to ensure economic efficiency and alignment with stakeholder and public needs before considering a broader rollout across Wales.

The findings of this study are expected to be of interest to services and researchers in Wales, as well as stakeholders in other countries seeking to evaluate and enhance data systems for the prevention of self-harm and interpersonal violence. Finalised results will be presented to local health services including EDs and mental health teams; related regional teams such as BCUHB public health, and Data, Insight and Intelligence Team; national programme teams (eg, Six Goals for Urgent and Emergency Care Programme team); and national and regional forums (eg, North Wales Regional Suicide and Self-Harm Prevention Forum). Findings will also be published as a peer-reviewed journal article to share learning more widely.

## Data Availability

Data sharing not applicable as no datasets generated and/or analysed for this study.
